# Synchronous Presentation of Intraperitoneal and Retroperitoneal Dedifferentiated Liposarcoma: A Case Report

**DOI:** 10.7759/cureus.110343

**Published:** 2026-06-06

**Authors:** Yousra Mhande, Laila Merghat, Amal Hajri, Driss Erguibi, Saad Rifki El Jai

**Affiliations:** 1 General Surgery, Faculty of Medicine and Pharmacy, Hassan II University, Casablanca, MAR; 2 General Surgery, Surgical Department of Cancerology and Liver Transplantation, Ibn Rochd University Hospital Center, Casablanca, MAR

**Keywords:** dedifferentiated liposarcoma, intraperitoneal liposarcoma, liposarcoma, mdm2, retroperitoneal liposarcoma

## Abstract

Liposarcomas (LPS) are a group of tumors with different subtypes, some of which are more aggressive. They most often arise in the retroperitoneum, while occurrence within the peritoneal cavity is uncommon. Symptoms are usually nonspecific. We report the case of a combined intraperitoneal and retroperitoneal dedifferentiated LPS (DDLPS), which presented significant diagnostic and therapeutic challenges.

We report the case of a 63-year-old woman with a history of recurrent retroperitoneal LPS, who presented to our department with right hypochondriac discomfort and progressive abdominal distention. Clinical and imaging examinations revealed bilateral masses: a left intraperitoneal mass and a right retroperitoneal mass. The patient underwent cytoreductive surgery involving the resection of the intraperitoneal mass, while the retroperitoneal tumor was deemed unresectable due to the invasion of the inferior vena cava. Histopathological analysis confirmed the diagnosis of DDLPS. Follow-up examinations showed a stable clinical condition.

Due to the rarity of intraperitoneal DDLPS, this case contributes valuable data to help improve understanding and support the development of standardized guidelines for its management. Additionally, it illustrates the challenges of treating complex recurrent retroperitoneal LPS and underscores the critical role of molecular murine double minute 2 (MDM2) analysis in diagnosis.

## Introduction

Accounting for less than 1% of adult malignancies, sarcomas are rare tumors, among which liposarcomas (LPS) represent the most common histologic subtype [1]. They are most frequently located in the retroperitoneum, where they often reach considerable size before detection and pose challenges for complete surgical resection due to the involvement of adjacent organs [1,2].

In contrast, intraperitoneal dedifferentiated LPS (DDLPS), arising from the mesentery or peritoneal cavity, is far less common, with an unknown incidence, as it has only been reported in isolated case studies. These tumors tend to present with earlier abdominal symptoms [3].

We report a rare case of a newly identified intraperitoneal LPS occurring alongside a late recurrent retroperitoneal lesion, highlighting the surgical challenges of multifocal disease and the critical role of molecular confirmation when immunohistochemistry is inconclusive.

## Case presentation

We report the case of a 63-year-old woman with diabetes mellitus and hypertension. Her surgical history included a resection of DDLPS with right radical nephrectomy in 2012, followed by adjuvant chemotherapy, and excision of her first recurrence in 2019, which resulted in positive surgical margins.

The patient presented to our department with a six-month history of right hypochondrial heaviness, progressive abdominal distension, and deterioration of her general condition manifested by progressive weight loss and asthenia, without nausea, vomiting, bowel transit disorders, jaundice, or dyspnea.

Physical examination revealed a soft, painless, and mobile mass in the left iliac fossa, extending to the left hypochondrium. A second large, hard, painless mass in the right hypochondrium was identified, mobile superficially but fixed deeply, extending to the epigastric region and right flank. Digital rectal examination revealed a firm bulge in the pouch of Douglas. Routine blood tests were unremarkable.

Abdominal computed tomography (CT) scan (Figure 1) and magnetic resonance imaging (MRI) (Figure 2) demonstrated an intraperitoneal tumor on the left side measuring 85 × 115 mm and extending vertically over 26 cm, with regular contours, an oblong hypodense appearance, and mild heterogeneous contrast enhancement. A second large mass was identified in the right renal fossa with both intraperitoneal and retroperitoneal extension. This mass displayed mixed solid and cystic components with mild contrast enhancement. Measuring 145 × 118 × 184 mm, it compressed the duodenopancreatic block and hepatic hilum, causing mild upstream dilatation of the intrahepatic bile ducts. These imaging findings suggested left-sided intraperitoneal and right-sided retroperitoneal LPS, consistent with the patient's known tumor pathology.

**Figure 1:**
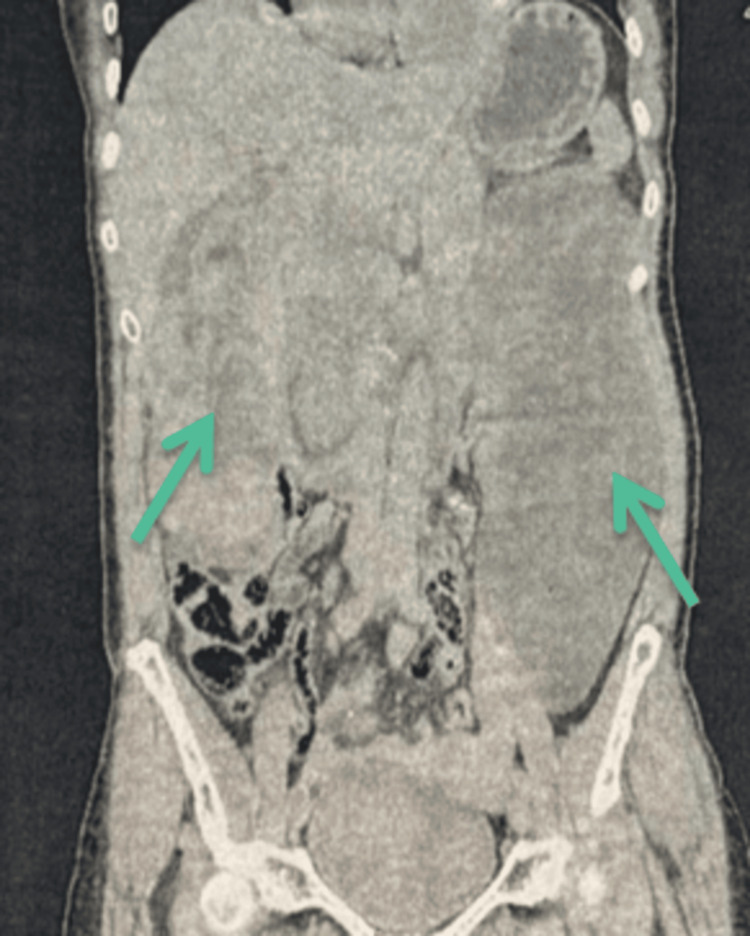
Coronal computed tomography scan of the abdomen showing bilateral abdominal masses (arrows).

**Figure 2:**
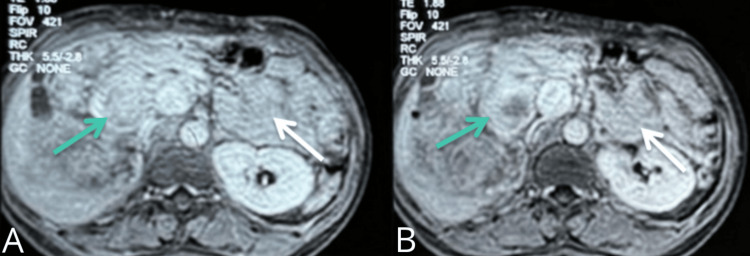
(A and B) Axial abdominal MRI demonstrating bilateral masses (arrows). MRI demonstrating bilateral abdominal masses: a left intraperitoneal liposarcoma (white arrow) and a right retroperitoneal liposarcoma (green arrow). MRI: magnetic resonance imaging

The case was discussed at a multidisciplinary meeting, and cytoreductive surgery was planned. Surgical exploration allowed the resection of the left mesenteric mass, which was in contact with the first jejunal loop and the inferior mesenteric vein (Figure 3 and Figure 4). The specimen weighed 1032 g and measured 30 cm (Figure 5). Dissection of the second mass, closely related to the right colon, gallbladder, duodenum (D1-D2), and hepatic pedicle, revealed intraoperatively that it encompassed the inferior vena cava and abdominal aorta, making dissection unsafe; therefore, therapeutic abstention was decided.

**Figure 3:**
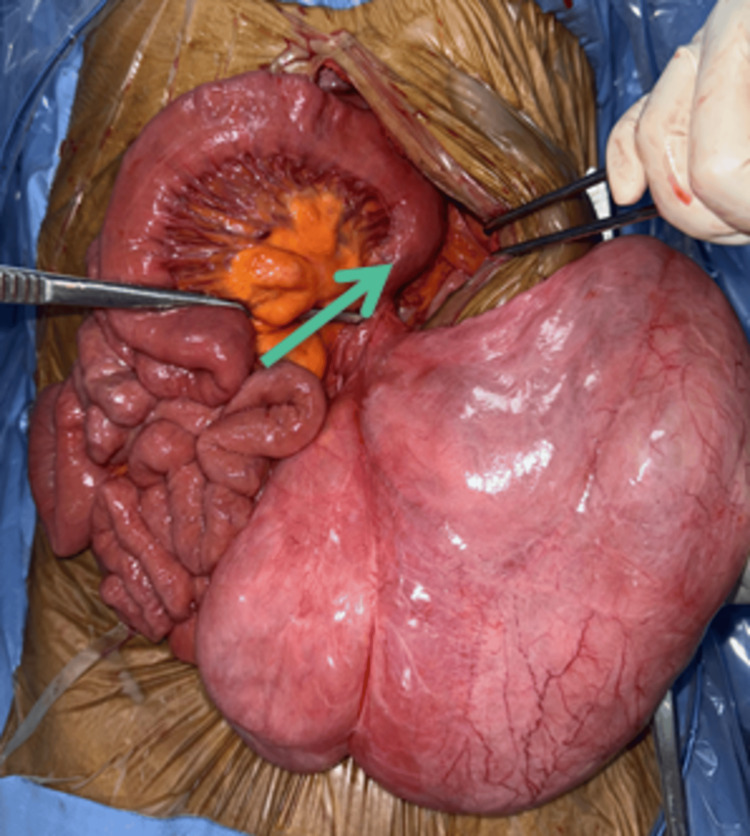
Contact between the intraperitoneal liposarcoma and the first jejunal loop (arrow).

**Figure 4:**
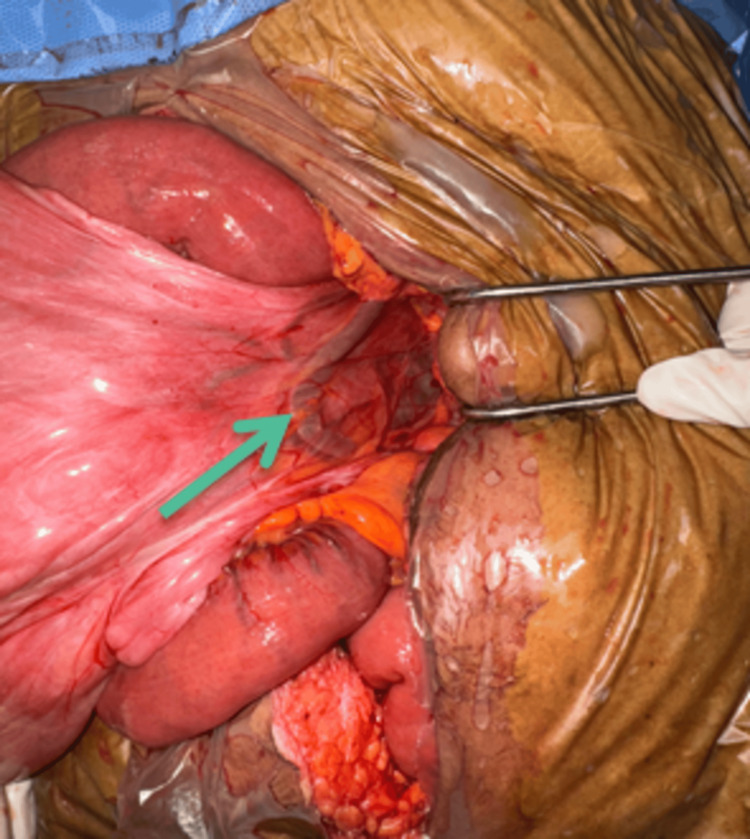
Contact between the intraperitoneal liposarcoma and the inferior mesenteric vein (arrow).

**Figure 5:**
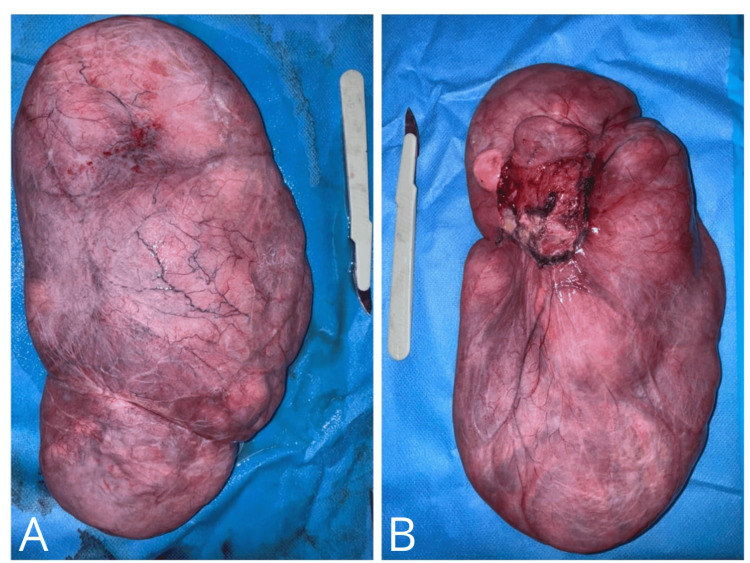
(A and B) Specimen of the intraperitoneal liposarcoma.

The hospital stay was complicated by diabetic ketoacidosis, which was successfully managed without an identified infectious source.

Histopathological examination revealed an encapsulated solid-cystic mass without capsular rupture, composed of stellate and spindle cells within a myxoid stroma, showing hyperchromatic nuclei with occasional prominent nucleoli and no necrosis. The mitotic index was 3 mitoses/2 mm². Murine double minute 2 (MDM2) immunohistochemistry was negative, while fluorescence in situ hybridization (FISH) demonstrated MDM2 gene amplification, confirming the diagnosis of DDLPS.

The patient was scheduled for chemotherapy and discharged eight days after surgery for follow-up. A CT scan performed four months postoperatively, following five courses of chemotherapy, showed that the retroperitoneal mass remained stable, with no signs of secondary lesions in the thoracic region. Given that the patient had been managed with palliative intent throughout the disease course, radiotherapy was not recommended based on the benefit-risk balance.

## Discussion

LPS represents the most frequent form of soft tissue sarcoma, accounting for approximately 45% of cases in the retroperitoneal area [4]. However, fewer than 90 cases of LPS originating within the intraperitoneal compartment, including the small bowel mesentery, greater omentum, mesocolon, or porta hepatis, have been reported, making this location quite rare [3]. These tumors typically develop around the sixth decade of life in both intraperitoneal and retroperitoneal locations. Reported cases of intraperitoneal LPS show a higher incidence in males, whereas retroperitoneal cases occur equally in both sexes [3,5].

LPS can be classified into four main histological subtypes: myxoid LPS (MLPS), pleomorphic LPS (PLPS), well-differentiated LPS (WDLPS), and DDLPS [6]. Each subtype is distinguished by its histomorphologic features and genetic alterations. Moreover, they exhibit different clinical behaviors, highlighting the importance of accurate subclassification [7]. Molecular testing for MDM2 significantly contributes to the precise subtyping of LPS [8].

Intraperitoneal and retroperitoneal LPS often grow silently, causing no specific symptoms until advanced stages. Patients with intraperitoneal LPS may notice abdominal fullness or pain, while retroperitoneal tumors can cause nonspecific symptoms such as abdominal discomfort, early satiety, or leg swelling [2,9].

A CT scan is usually the first imaging test performed for this disease. WDLPS and DDLPS often have distinctive features that make them visible on both CT and MRI. Contrast-enhanced CT is typically used for abdominal and retroperitoneal tumors, whereas MRI is especially useful for pelvic tumors or extremity lesions, when contrast cannot be administered, or when the tumor's relationship to nearby structures is unclear [5,10].

Complete surgical resection of the tumor is considered the primary treatment approach for both intraperitoneal and retroperitoneal LPS and is associated with improved surgical outcomes [4,9]. However, retroperitoneal DDLPS is challenging to treat due to its deep location, which is associated with a higher risk of local recurrence and disease-specific mortality. Additionally, intraperitoneal LPS tend to recur frequently when located in high-risk areas that limit the possibility of complete resection [3,4]. Outcomes are influenced by margin status, with R2 resections linked to poorer survival than R0 or R1 resections, and maintaining the specimen's integrity during surgery also impacts prognosis [11].

For patients with unresectable or metastatic DDLPS, anlotinib represents a therapeutic alternative. This oral multi-target tyrosine kinase inhibitor blocks vascular endothelial growth factor receptor (VEGFR), fibroblast growth factor receptor (FGFR), platelet-derived growth factor receptor (PDGFR), and c-Kit and has shown antitumor activity in soft tissue sarcomas and other cancers [12,13].

A meta-analysis by Albertsmeier et al. demonstrated that external beam radiotherapy improves overall survival in retroperitoneal soft tissue sarcomas and reduces local recurrence [14]. The role of adjuvant chemotherapy is limited because of the disease's resistance to conventional cytotoxic agents [4]. Several factors influence prognosis in this disease. Achieving negative microscopic surgical margins is a key predictor of local recurrence, and histologic grade, reflecting the level of tumor differentiation, remains a crucial determinant of disease course and outcome [15].

After surgery, follow-up is determined by sarcoma grade. Patients with low-grade tumors are typically followed with CT scans every 4-6 months for the first 3-5 years and then annually. In contrast, intermediate- and high-grade sarcomas require closer follow-up, with imaging every 3-4 months during the first 2-3 years, every six months until the fifth year, and once a year thereafter [16].

## Conclusions

Intraperitoneal LPS are rare tumors, whereas retroperitoneal LPS present significant treatment challenges. Synchronous intraperitoneal and retroperitoneal involvement represents an uncommon presentation of LPS. Histopathological evaluation combined with molecular detection of MDM2 amplification, particularly by FISH, plays a key role in confirming the diagnosis of LPS and represents an important diagnostic tool.

Due to their complexity, LPS require multidisciplinary management. Surgery remains the fundamental approach to therapy, as incomplete resection increases the risk of recurrence. Adjuvant therapy should be individualized according to the clinical context, and prolonged close follow-up is required, even in patients with stable disease.
